# Tunneling Currents in the Hyperbolic Phase Space

**DOI:** 10.3390/e26080639

**Published:** 2024-07-28

**Authors:** Ivan F. Valtierra, Andrei B. Klimov

**Affiliations:** Departamento de Física, Universidad de Guadalajara, Guadalajara 44420, Jalisco, Mexico; ivan.valtierra@academicos.udg.mx

**Keywords:** phase space, tunneling, quantum current

## Abstract

We introduce the quantum currents for quantum systems with an SU(1,1) dynamic symmetry group whose evolution is governed by a non-linear Hamiltonian possessing a continuous spectrum and apply them to the analysis of the tunneling dynamics on the hyperbolic phase space.

## 1. Introduction

One of the most representative purely quantum effects is tunneling, which is ordinarily considered a penetration through a potential barrier that cannot be classically overcome [1,2,3,4,5]. This common view of quantum dynamics is appropriate for the description of quantum systems representable in coordinate-momentum form, which is algebraically described in terms of the Heiseiberg–Weyl (HW) algebra.

It is important to note that the coordinate-momentum form, while suitable for simple quantum systems possessing the HW symmetry, is not applicable for those with the highest symmetries. This limitation underscores the need for alternative frameworks like the phase-space approach [6,7] to understand and describe complex quantum phenomena like tunneling deeply. The advantage of the phase-space description of quantum dynamics is not only that it can be visualized, at least in low-dimensional phase-space manifolds, but also that the evolution equation can be represented in Liuvillian-type form, which allows for convenient analysis of the semiclassical limit, describing the evolution through a propagation along classical trajectories.

In the phase-space framework, a tunneling process can be seen as propagating through regions separated by classical separatrix. Each area is classically invariant and thus preserved during the semiclassical dynamics in phase space, which differs from the standard WKB approach in the configuration space (see e.g., [8,9] and references therein). The quantum evolution in phase space does not prevent crossing the classical separatrix and propagating through classically forbidden regions. It is worth noting that while the phase-space framework is just a valuable tool for analyzing the tunneling phenomenon in the configuration space [10,11,12,13,14], it becomes fundamental for quantum systems with the highest symmetries, as, e.g., spin-like systems described by the SU(2) group (see the recent review and references therein [15]), quantum parametric amplifier models described by the SU(1,1) group [16,17,18,19,20], and, in general, multilevel quantum systems with the SU(N) dynamic symmetry group [21,22].

The simplest form to map an operator $\hat{f}$ acting in the Hilbert space H of a quantum system, carrying an irreducible representation of a dynamic group *G*, into distributions in the corresponding classical phase-space M is achieved by its projection into the set of coherent states {|ζ〉, ζ∈M} labeled by points of the manifold M where *G* acts transitively [23,24,25]. As a result, the Husimi Q-symbols are obtained [26],

(1) $$Q_{f}\left(\zeta \right)=\langle \zeta |\hat{f}|\zeta \rangle .$$

The Q-symbol of the density matrix $\hat{\varrho }$ is a positive and smooth function (the so-called Q-function), $Q_{\varrho }\left(\zeta \right)\equiv Q\left(\zeta \right)$.

The evolution equation for the Q-function is obtained by applying the map (1) to the Schrodinger equation and can be formally represented as

(2) $$\partial_{t}Q\left(\zeta ,t\right)\;=L_{H}\left(\zeta \right)Q\left(\zeta ,t\right)\;,$$

where $L_{H}\left(\zeta \right)$ is a differential operator whose order depends on the degree of the Hamiltonian on the generators of the dynamic symmetry group. The above equation contains, in general, higher-order derivatives that allow us to describe the quantum dynamics entirely in the language of phase-space variables. In addition, Equation (2) can be represented in the form of a continuity equation on M:

(3) $$\begin{array}{ccc} \partial_{t}Q\left(\zeta ,t\right) & = & -\mathrm{div}J(\zeta ,t)\;, \end{array}$$

(4) $$\begin{array}{ccc} J(\zeta ,t) & = & J(\zeta )Q(\zeta ,t), \end{array}$$

where J(ζ) is a differential operator. The current J(ζ,t) describes the propagation of the initial distribution [14,27,28,29,30,31,32,33], and its phase-space configuration can be used for the detection of quantum features of the evolution.

The semiclassical dynamics corresponds to the limit when the operator $L_{H}\left(\zeta \right)$ is reduced to the Poisson brackets on M. In this approximation, the initial distribution evolves along the classical trajectories and behaves as a non-compressible liquid. For practical purposes, it is advantageous to begin with a state represented by a localized distribution when studying phase-space tunneling. Thus, a Q-distribution initially localized inside a given dynamic region cannot propagate into another one during the semiclassical evolution. The semiclassical current operator generated by the Hamiltonian flow, $J\left(\zeta \right)∼\nabla Q_{H}\left(\zeta \right)$, is converted into a multiplicative coordinate-dependent factor.

The Q-function, by design, is exponentially small during the evolution in classically forbidden (for a given initial state) regions. In these regions, the magnitude of the classical Hamiltonian is significantly larger than both the average value and the fluctuation of the quantum Hamiltonian in the initial state. As a result, the tunneling dynamics in phase space is observed as the sudden splitting of an initial localized distribution into well-separated pieces, which is followed by a recovery of a deformed but still well-localized cumulus. The quantum current captures the qualitative features of separatrix crossing, and its appropriate marginals can be utilized to determine the phase-space tunneling time.

In the present paper, we introduce the quantum currents for quantum systems with the SU(1,1) dynamic symmetry group in the hyperbolic phase space [34]. We will analyze the evolution governed by a quadratic Hamiltonian whose phase space becomes divided by a separatrix into several dynamical regions containing hyperbolic classical trajectories at a critical value of the system’s parameter. The transition through classically forbidden regions will be discussed in terms of the spatial distributions of the quantum currents. It is worth noting that the evolution of unbound Hamiltonians and an appropriate map into the hyperbolic phase space is a tricky problem requiring a careful numerical analysis (see Appendix A).

In quantum-optical applications, quantum systems with SU(1,1) symmetry typically appear in the description of coupled evolution of two-mode quantized field modes [35,36,37,38,39,40]. From the physical perspective, the phase-space tunneling corresponds to a deeply non-classical regime of the dynamics of certain observables, e.g., the difference of excitation numbers of each mode. We will put these two processes in correspondence and relate the characteristic timescales.

In Section 2, we outline the main concepts of the SU(1,1) phase-space approach. In Section 3, we analyze the semiclassical and quantum evolution, governed by a non-linear Hamiltonian, of the Q-function in the hyperbolic phase space and describe the tunneling process in terms of quantum currents. A short discussion of applications to quantum optical systems is present in Section 4.

## 2. Q-Function on the Hyperboloid

Let us consider a system with an SU(1,1) dynamic symmetry group, in which generators ${\hat{K}}_{j}$, j=0,1,2 from the su(1,1) algebra satisfy the commutation relations,

(5) $$\left[{\hat{K}}_{1},{\hat{K}}_{2}\right]=-i{\hat{K}}_{0},\;\;\;\;\;\;\;\left[{\hat{K}}_{2},{\hat{K}}_{0}\right]=i{\hat{K}}_{1},\;\;\;\;\;\;\;\left[{\hat{K}}_{0},{\hat{K}}_{1}\right]=i{\hat{K}}_{2}.$$

The eigenvalues of the Casimir operator

(6) $$\hat{C}={\hat{K}}_{0}^{2}-{\hat{K}}_{1}^{2}-{\hat{K}}_{2}^{2},$$

$c_{k}=k\left(k-1\right)$ label the irreducible representations of the group. The Hilbert space H that carries an irrep labeled by the Bargman index $k=\frac{1}{2},1,\frac{3}{2},2,\ldots$, corresponding to the positive discrete series, is spanned by the eigenstates of the ${\hat{K}}_{0}$ operator,

(7) $${\hat{K}}_{0}|k,k+m\rangle =\left(k+m\right)|k,k+m\rangle \;,\;m=0,1,\ldots \;,$$

where |k,k〉 is the lowest state of the representation, which is defined by ${\hat{K}}_{-}|k,k\rangle =0$, ${\hat{K}}_{\pm }={\hat{K}}_{1}\pm i{\hat{K}}_{2}$.

A set of coherent states [23],

(8) $$|n\rangle =\cosh^{-2k}\frac{\tau }{2}\sum_{m=0}^{\infty }{\left[\frac{\Gamma (m+2k)}{m!\Gamma (2k)}\right]}^{1/2}e^{-i\phi m}\tanh^{m}\frac{\tau }{2}|k,k+m\rangle \;,\;$$

is labeled by the coordinates (τ,ϕ) of hyperbolic Bloch vectors in the upper sheet of the two-sheet hyperboloid n·n=1,

(9) $$n={\left(\cosh\tau ,\sinh\tau \cos\phi ,\sinh\tau \sin\phi \right)}^{⊤}\;,$$

and resolve the identity according to

(10) $$\hat{I}=\frac{2k-1}{\pi }\int d^{2}\;n|n\rangle \langle n|\;,$$

where $d^{2}n=\frac{1}{4}\sinh\tau d\tau d\phi$ is the invariant measure. This hyperboloid can be considered as a phase space of our quantum system.

The SU(1,1) Q-function defined as

(11) $$Q\left(n\right)=\langle n|\hat{\rho }|n\rangle ,\;\frac{2k-1}{\pi }\int d^{2}\;nQ\left(n\right)=1,$$

is a positive normalized distribution on the hyperboloid that contains the same information as contained in the density matrix.

The Q-function of a coherent state $|n_{0}\rangle$ is

(12) $$\begin{array}{ccc} |\langle n|n_{0}\rangle {|}^{2} & = & {\left(\frac{1+n\cdot n_{0}}{2}\right)}^{-2k}, \end{array}$$

(13) $$\begin{array}{ccc} n\cdot n_{0} & = & \cosh\tau \cosh\tau_{0}-\cos\left(\phi -\phi_{0}\right)\sinh\tau \sinh\tau_{0}, \end{array}$$

which is represented as a smooth localized distribution on the hyperboloid.

## 3. Tunneling Currents on the Hyperboloid

We will focus on a particular case of the Lipkin–Meshkov–Glick model on the hyperboloid described by the following Hamiltonian quadratic on the SU(1,1) generators,

(14) $$\hat{H}={\hat{K}}_{0}+\frac{\mu }{k}{\hat{K}}_{2}^{2}.$$

The corresponding classical Hamiltonian is given by the Q-symbol of (14),

(15) $$Q_{H}\left(n\right)=\cosh\tau +\mu \left(2k+1\right)\sinh^{2}\tau \sin^{2}\phi =H_{cl}.$$

The structure of the classical orbits, defined by the Hamiltonian equations of motion,

(16) $$\begin{array}{ccc} \dot{\tau } & = & -\mu \sinh\tau \sin2\phi , \end{array}$$

(17) $$\begin{array}{ccc} \dot{\phi } & = & 1+2\mu \cosh\tau \sin^{2}\phi . \end{array}$$

is fundamentally different for −1/2<μ<0 and μ<−1/2.

**Figure 1:**
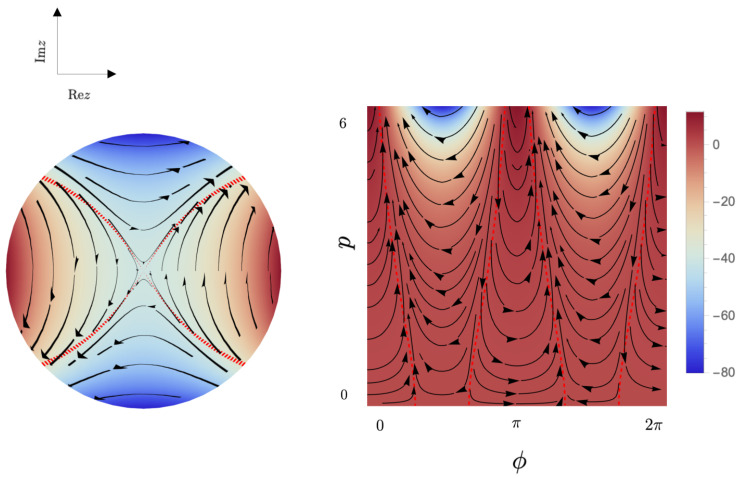
The phase portrait for the classical Hamiltonian (15) k=10,μ=−1. (**Left**): Density plot on the unit disc $D:=\{z=\tanh\frac{\tau }{2}e^{-i\phi }:|z|<1\}$; (**Right**): Density plot on the plane p−ϕ, where p=coshτ−1. The red dashed lines are the classical separatrices.

In the latter case, μ<−1/2, $Q_{H}\left(\tau ,\phi \right)$ acquires a form of a two-valley potential, where the minima of the valley are located at $\phi_{\min}=\pi /2,3\pi /2$ and separated by crests whose maxima are at $\phi_{\max}=0,\pi$. The separatrices

(18) $$\tau ={\mathrm{arccosh}}^{-1}\left(-\frac{1}{2\mu }\csc^{2}\phi \right),$$

divide the phase space into four classically invariant regions along with the corresponding families of hyperbolic trajectories, as shown in Figure 1. This situation corresponds to the presence only of continuous components in the spectrum of (14).

### 3.1. Semiclassical Evolution

On the phase-space language, the Hamiltonian (15) generates a semiclassical evolution of the Q-function according to the Liouvillian equation,

(19) $$\frac{\partial Q^{cl}\left(n,t\right)}{\partial t}={\left\{Q^{cl}\left(n,t\right),Q_{H}\left(n\right)\right\}}_{P}=-\mathrm{div}J^{cl}\left(n,t\right)\;,$$

in which ${\left\{,\right\}}_{P}$ are the Poisson brackets on the hyperboloid,

$${\left\{f,g\right\}}_{P}=\frac{1}{\sinh\tau }\left(\partial_{\phi }f\partial_{\tau }g-\partial_{\tau }f\partial_{\phi }g\right).$$

Thus, every point of the initial distribution Q(n,t=0) evolves along the corresponding classical trajectory n(t):

$$Q^{cl}\left(n,t\right)=Q^{cl}\left(n\left(t\right)\right).$$

**Figure 2:**
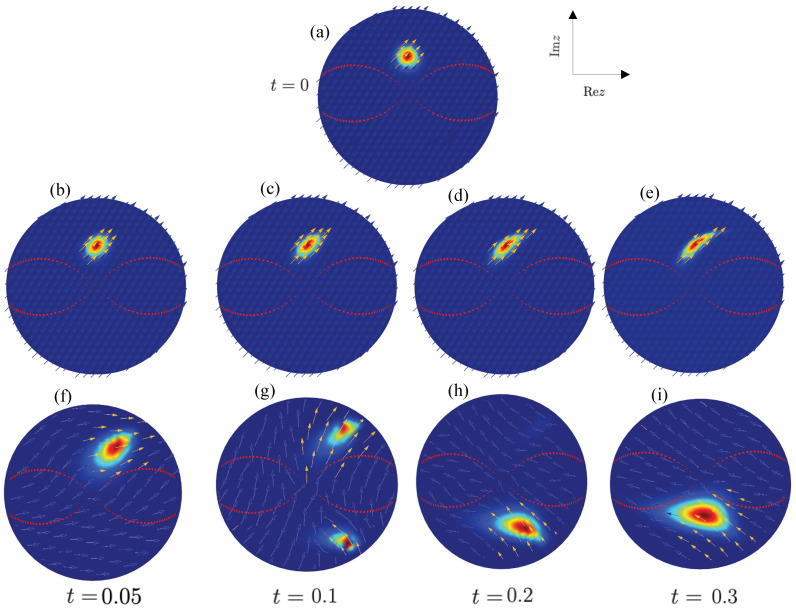
Snapshots of the Q(n,t)-function corresponding to the initial coherent state (**a**), $|\tau_{0}=1.5$, $\phi_{0}=\pi /2\rangle$: the classical dynamics (**b**–**e**) and the quantum dynamics (**f**–**i**).

In other words, the evolution of the Q-function is generated by the Hamiltonian currents,

$$\;J^{cl}\left(n,t\right)=J^{cl}\left(n\left(t\right)\right)=\left(J_{\phi }^{cl}\left(t\right),J_{\tau }^{cl}\left(t\right)\right),$$

having in the particular case of the Hamiltonian (15) the form,

(20) $$\begin{array}{ccc} J_{\phi }^{cl}\left(t\right) & = & -Q_{\rho }\left(n\left(t\right)\right)\partial_{\tau }Q_{H}\left(n\right)=\frac{\mu }{2}\;\sinh\tau \sin2\phi Q\left(n\left(t\right)\right), \end{array}$$

(21) $$\begin{array}{ccc} J_{\tau }^{cl}\left(t\right) & = & \frac{1}{\sinh\tau }Q_{\rho }\left(n\left(t\right)\right)\partial_{\phi }Q_{H}\left(n\right)=-\left(\sinh\tau +\mu \sin^{2}\phi \sinh2\tau \right)Q\left(n\left(t\right)\right), \end{array}$$

generate a motion of a distribution initially localized inside one of the invariant regions toward the corresponding separatrix. In Figure 2 (lower panel), we plot the evolution of the Q-distribution corresponding to a coherent state (8) initially centered at $n_{0}=\left(\tau_{0}=1.5,\phi_{0}=\pi /2\right)$ inside a “potential valley”. At this point, it is fulfilled $\sigma_{H}\left(n_{0}\right)\ll H_{cl}\left(\tau ,\phi =0,\pi \right)-H_{cl}\left(n_{0}\right)$, where $\sigma_{H}^{2}\left(n_{0}\right)=\langle n_{0}|{\hat{H}}^{2}|n_{0}\rangle -(\langle n_{0}|\hat{H}|n_{0}{\rangle \;)}^{2}$. The amplitude of the Q-distribution, being put at $n_{0}$, is significantly smaller than the maxima of the surrounding crests of the potential $Q_{H}\left(\tau ,\phi =0,\pi \right)$, and the dispersion of $Q_{\rho }\left(n\right)$ is essentially narrower than the average width of the valley. Thus, the value of the initial Q-function in classically prohibited regions (in the vicinity of the potential crests) is exponentially small, and all meaningful dynamics are concentrated inside the initial valley. The semiclassical evolution, consisting of a propagation of distinct points of the distribution along the corresponding classical trajectories, leads to displacements and deformations of the initial Q-function in the classically accessible region. The classical currents (20) and (21) indicate the direction of the propagation and the distortion of the initial distribution. Obviously, no transition into another classically permitted region (the potential valley centered at 3π/2) happens. The deformation of the distribution becomes very strong when it becomes close to the separatrix.

### 3.2. Quantum Evolution

The quantum phase-space dynamics is drastically different from the classical one. The evolution equation for the Q-function is a second-order partial differential equation [41], which can be conveniently represented in a Liouville-like form,

(22) $$\partial_{t}Q\left(n,t\right)={\left\{n_{0},Q\left(n,t\right)\right\}}_{p}+\frac{\mu }{k}{\left\{n_{2},G_{2}Q\left(n,t\right)\right\}}_{p},$$

where $G_{2}$ is a first-order differential operator

$$G_{2}=2k\sinh\tau \sin\phi +\frac{1}{\sinh\tau }\left(\cos\phi \partial_{\phi }+\frac{1}{2}\sinh2\tau \sin\phi \partial_{\tau }\right).$$

The dynamics of Q(n,t) is induced by the quantum currents,

$$\begin{array}{ccc} J_{\tau }\left(n,t\right) & = & \frac{\mu }{2k}\cos\phi \;G_{2}Q\left(n,t\right)\\ J_{\phi }\left(n,t\right) & = & -\left(\sinh\tau +\frac{\mu }{k}\sin\phi \cosh\tau G_{2}\right)Q\left(n,t\right), \end{array}$$

that not only indicate the directions of deformations of Q(n,t) but also describe its propagation through areas separated by classical separatrices.

The snapshots of Q(n,t) corresponding to the initial coherent state $|\tau_{0}=1.5$, $\phi_{0}=\pi /2\rangle$ along with the distribution of the currents J(n,t) are plotted in Figure 2. The semiclassical evolution of $Q_{\rho }\left(n\left(t\right)\right)$ originally located in the upper valley, Figure 2a, is reduced to a deformation of the initial distribution generated by the classical currents (20) and (21) corresponding to a propagation along the trajectories (16) and (17); see Figure 2b–e. The quantum phase-space dynamics is significantly different; see Figure 2f–i. In contrast to classical evolution, quantum currents cause not only a distortion of the Q-function but also a significant displacement of its center from the original location in a short time. Despite an apparent translation of the maximum of the distribution in the direction of growth $\left|z\right|=\tanh\frac{\tau }{2}$ (mainly explained by the form of mapping the hyperboloid into a unit disc), the phase-space coordinate τ does not reach large values during the evolution, as discussed in Section 3. Actually, it oscillates around its initial value τ∼1.5 with an amplitude ∼0.17. For longer times, the distribution starts tunneling through the potential barrier. This process is seen as splitting a distribution into two separated peaks, where one piece is maintained in the initial valley, and the other emerges in another classically allowed valley. No direct movement of the Q-function through a potential crest, which is a classically forbidden region, is detected. The current directions and their intensity distribution indicate the main paths of the state transfer from one valley to another. The phase-space Q-currents are small but still detectable when the distribution “tunnels” through the classically forbidden area despite the magnitude of the Q-distribution, which is practically negligible. It is interesting to note that the distribution approximately keeps its localized form after the first act of tunneling. The general character of the phase-space evolution is preserved for longer times, except that the distribution becomes more and more delocalized.

The normalized integral azimuthal current

(23) $$I\left(\phi ,t\right)=\frac{1}{k}\int_{0}^{\infty }d\tau \sinh\tau J_{\phi }\left(n,t\right)\;,$$

provides valuable information about the tunneling process, indicating the time intervals of the maximum and minimum azimuthal flows at all angles.

**Figure 3:**
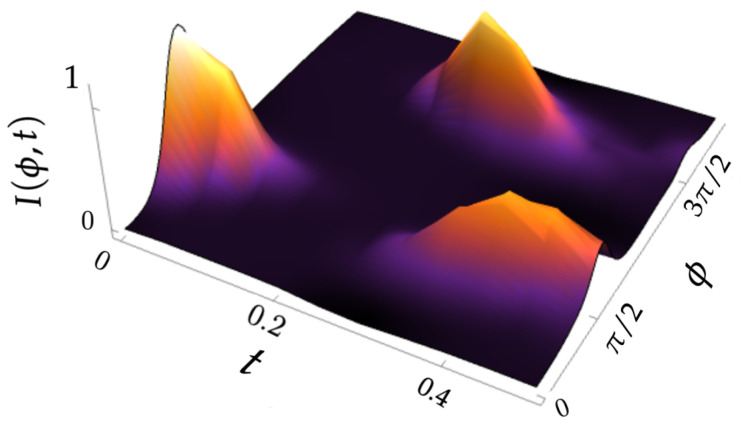
The integral azimuthal Husimi current (23) up to times corresponding to the returning of the Q-function in the original classically admissible valley for the initial coherent state $|\tau_{0}=1.5$, $\phi_{0}=\pi /2\rangle$.

In Figure 3, we plot I(ϕ,t) for times corresponding to two acts of tunneling, i.e., when the distribution returns to the initial valley. As expected, I(ϕ,t) is initially concentrated at ϕ=π/2 and is always almost zero at the angles corresponding to classically forbidden areas. The transfer of the distribution from the original to another potential valley is manifested in the appearance of the integral current localized at the minimum of the valley at ϕ=3π/2. The time interval between these maxima can be considered as a tunneling time [42,43] in the phase space. A less pronounced third maximum reflects a process of the delocalization of the distribution in the course of evolution.

## 4. Discussion

**Figure 4:**
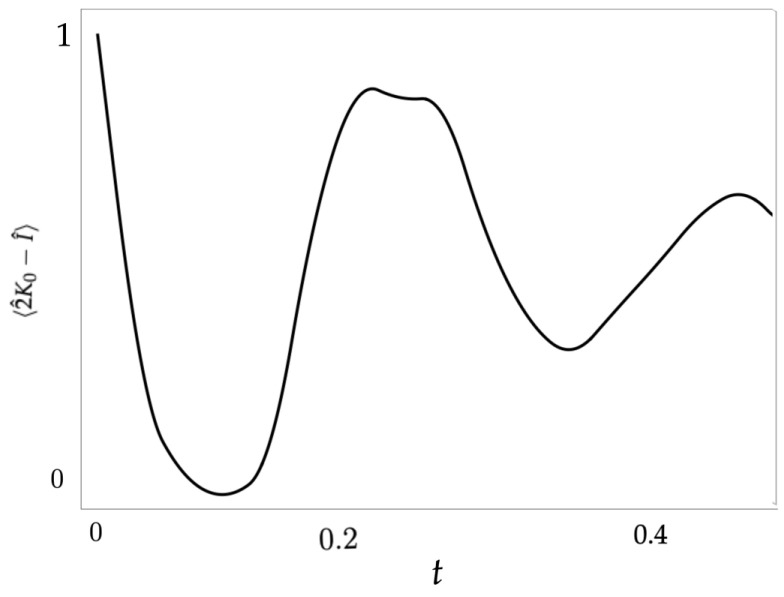
The evolution of the normalized total number of excitations $N\left(t\right)/2k=\langle 2{\hat{K}}_{0}-\hat{I}\rangle /2k$ for the Hamiltonian (14) and the initial coherent state $|\tau_{0}=1.5,\phi_{0}=\pi /2\rangle$.

In quantum optics, the su(1,1) algebra appears in the description of the non-degenerate parametric amplifier, where

(24) $${\hat{K}}_{+}={\hat{a}}^{†}{\hat{b}}^{†},\;{\hat{K}}_{-}=\hat{a}\hat{b},\;{\hat{K}}_{0}=\frac{1}{2}\left({\hat{a}}^{†}\hat{a}+{\hat{b}}^{†}\hat{b}+\hat{I}\right),$$

and where $\hat{a}$ and $\hat{b}$ are the boson operators. The coherent states (8), physically generated in quantum parametric processes, form an overcomplete basis in each Hilbert space with a fixed difference Δn of excitations between the modes *a* and *b*. The SU(1,1)-irreducible subspaces are labeled by $k=\frac{1}{2}\left(1+|\Delta n|\right)$. Non-linear Hamiltonians similar to (14) describe the dynamics of coupled field modes. One of the physically relevant observables is the total number of excitations *N*, corresponding to the average value of $2{\hat{K}}_{0}-\hat{I}$. In Figure 4, we plot the evolution of *N* for the Hamiltonian (14) and the initial coherent state $|\tau_{0}=1.5$, $\phi_{0}=\pi /2\rangle$. The oscillatory behavior of N(t) is in accordance with the phase-space dynamics; see Figure 2. It is worth noting that the phase-space variable $\cosh\tau (t)=\langle {\hat{K}}_{0}\left(t\right)\rangle /k$ does not reach significant values during the evolution, which is physically explained by a largely “detuned” interaction dynamics of the quantized modes described by the Hamiltonian (14). The maximum of the osculation observed at t≈0.25 corresponds to the complete “tunneling” of the distribution between classically admissible valleys. Taking into account that the tunneling phenomenon is essentially a quantum effect, one can attribute the same nature to the process of photon generation in the parametric dynamics governed by (14).

Thus, the quantum currents J(n,t) are a valuable tool for analyzing the evolution in the hyperbolic phase space. The spatial distribution of the quantum current allows one to visualize the main directions of both semiclassical and quantum propagations of the distribution. The marginal current distribution (23) can be used for the determination of the phase-space tunneling time. The tunneling time defined in this way determines the characteristic period of the deeply non-classical evolution of physical observables related to the system’s Hamiltonian.

An advantage of using the Q-function is its positivity, which allows us to consider it a “true” probability distribution and observe its delocalization (splitting) in the phase space related to the tunneling process. However, the Q-representation does not always thoroughly exhibit the quantum interference pattern. An attractive possibility for studying phase-space tunneling would be to analyze the currents associated with the Wigner distribution for the SU(1,1) group. A faithful Wigner distribution [34] reveals a smooth transition to the classical regime in the limit of a large representation index [41]. In addition, one could expect an involved quantum interference picture and a non-vanishing Wigner flow in the classically forbidden areas. This problem will be addressed in future work.

## Data Availability

The original contributions presented in the study are included in the article, further inquiries can be directed to the corresponding author.

## Appendix A

In this Appendix, we describe a procedure used for the numerical analysis of the evolution generated by the Hamiltonian (14) and the mapping into the phase-space Q-distribution.

Following [44], we denote the eigenstates of ${\hat{K}}_{2}$ as

(A1) $${\hat{K}}_{2}|\lambda ,k\rangle =\lambda |\lambda ,k\rangle ,$$

and introduce Hermitian parabolic operators

$${\hat{J}}_{\pm }={\hat{K}}_{1}\pm {\hat{K}}_{0},$$

obeying the commutation relations

(A2) $$\left[{\hat{K}}_{2},{\hat{J}}_{\pm }\right]=\pm i{\hat{J}}_{\pm },\;\left[{\hat{J}}_{+},{\hat{J}}_{-}\right]=2i{\hat{K}}_{2}.$$

Then, the states ${\hat{J}}_{\pm }|\lambda \rangle$ are also eigenstates of ${\hat{K}}_{2}$ with complex eigenvalues,

$${\hat{K}}_{2}({\hat{J}}_{\pm }|\lambda ,k\rangle )=\left(\lambda \pm i\right)({\hat{J}}_{+}|\lambda ,k\rangle ).$$

Now, one introduces normalizable states in the form of expansion

$$|\phi \rangle =\int_{-\infty }^{\infty }d\lambda \phi \left(\lambda \right)|\lambda ,k\rangle ,$$

such that the states ${\hat{J}}_{\pm }|\phi \rangle$ are also normalizable.

In the *x*-representation defined as

(A3) $$\psi \left(x\right)=\frac{1}{\sqrt{2\pi }}\int_{-\infty }^{\infty }e^{i\lambda x}\phi \left(\lambda \right)d\lambda ,$$

the generators ${\hat{K}}_{2}$ and ${\hat{J}}_{\pm }$ have the following non-symmetric realization,

$$\begin{array}{ccc} {\hat{K}}_{2} & = & -i\partial_{x},\\ {\hat{J}}_{+} & = & e^{-x}\left(-\partial_{x}^{2}+\partial_{x}+k\left(k+1\right)\right),\\ {\hat{J}}_{-} & = & -e^{x}. \end{array}$$

The Schrödinger equation for the Hamiltonain (14) in the *x*-representation acquires the form

(A4) $$i\partial_{t}\psi \left(x,t\right)=\frac{1}{2}\left(e^{x}+e^{-x}k\left(k+1\right)-e^{-x}\partial_{x}\left(\partial_{x}-1\right)\right)\psi \left(x,t\right)-\frac{\mu }{k}\partial_{x}^{2}\psi \left(x,t\right).$$

To find the appropriate form of the initial condition for (A4), we expand the coherent state (8) in the basis (A1),

(A5) $$|n_{0}\rangle \equiv |\zeta_{0}=e^{-i\phi_{0}}\tanh\frac{\tau_{0}}{2}\rangle =\int_{0}^{\infty }d\lambda \zeta_{0}\left(\lambda \right)|\lambda ,k\rangle ,$$

where

$$\zeta_{0}\left(\lambda \right)=\frac{\left(1-\right|\zeta_{0}{{|}^{2})}^{k}}{{\left(1-\zeta_{0}^{2}\right)}^{k}}\frac{2^{2k-3/2}}{\pi \sqrt{\Gamma (2k)}}\Gamma \left(\frac{k-i\lambda }{2}\right)\Gamma \left(\frac{k+1+i\lambda }{2}\right){\left(\frac{1+\zeta_{0}}{1-\zeta_{0}}\right)}^{i\lambda },$$

which allows to recast the initial coherent state in the *x*-representation (A3) in the form of the Barnes integral:

(A6) $$\begin{array}{ccc} \psi_{0}\left(x,0\right) & = & \frac{1}{\sqrt{2\pi }}\int_{-\infty }^{\infty }e^{i\lambda x}\zeta_{0}\left(\lambda \right)d\lambda =A_{k}\left(\zeta_{0}\right)\frac{2^{k}e^{kx}}{{\left(1+{\left(e^{x}\frac{1+\zeta_{0}}{1-\zeta_{0}}\right)}^{2}\right)}^{k+1/2}}, \end{array}$$

(A7) $$\begin{array}{ccc} A_{k}\left(\zeta_{0}\right) & = & \frac{\left(1-\right|\zeta_{0}{{|}^{2})}^{k}\Gamma \left(k+1/2\right)2^{k+1/2}}{{\left(1-\zeta_{0}\right)}^{2k}\sqrt{2\pi }\sqrt{\Gamma (2k)}} \end{array}$$

The Q-function in the basis (A1) is

$$Q\left(\zeta \right)=|\langle \zeta |\zeta_{0}\rangle {|}^{2}={\left|\int_{-\infty }^{\infty }d\lambda \zeta^{*}\left(\lambda \right)\zeta_{0}\left(\lambda \right)\right|}^{2}.$$

Representing the solution of (A4) as a Fourier transform

$$\psi \left(x,t\right)=\frac{1}{\sqrt{2\pi }}\int_{-\infty }^{\infty }d\lambda e^{i\lambda x}\zeta_{0}\left(\lambda ,t\right)$$

we arrive at the following form of the evolved Q-function,

(A8) $$Q\left(\zeta ,t\right)=\frac{1}{2\pi }{\left|\int_{-\infty }^{\infty }d\lambda \int_{-\infty }^{\infty }dx\;e^{-i\lambda x}\psi \left(x,t\right)\zeta^{*}\left(\lambda \right)\right|}^{2}$$

where

$$\zeta^{*}\left(\lambda \right)=A_{k}^{*}\left(\zeta \right)\Gamma \left(\frac{k+i\lambda }{2}\right)\Gamma \left(\frac{k+1-i\lambda }{2}\right){\left(\frac{1+\zeta^{*}}{1-\zeta^{*}}\right)}^{-i\lambda }.$$

Computing the Barnes integral appearing in (A8), we finally obtain

$$Q\left(\zeta ,t\right)=\frac{{\left(1-|\zeta \right|}^{2}{)}^{2k}2^{4k}\Gamma {\left(k+1/2\right)}^{2}}{{\pi \Gamma \left(2k\right)|\left(1-\zeta \right)}^{2k}{|}^{2}}|\int_{-\infty }^{\infty }\psi^{*}\left(x,t\right)\frac{e^{kx}}{{\left(1+{\left[\frac{1+\zeta }{1-\zeta }\right]}^{2}e^{2x}\right)}^{k+1/2}}{|}^{2},$$

where ψ(x,t) is the solution of (A4) with the initial condition (A6).
